# Influence of the maternal rumen microbiome on development of the calf meconium and rumen microbiome^1^

**DOI:** 10.1093/tas/txaa136

**Published:** 2020-12-22

**Authors:** Kelly L Woodruff, Gwendolynn L Hummel, Kathleen J Austin, Travis L Smith, Hannah C Cunningham-Hollinger

**Affiliations:** 1 Department of Animal Science, University of Wyoming, Laramie, WY; 2 Laramie Research and Extension Center, University of Wyoming, Laramie, WY

## INTRODUCTION

The increasing demand for meat and milk products due to the rising population, coupled with limited resources, requires increased efficiency of livestock. The microbiome within the rumen allows ruminant species to convert low-quality forages into volatile fatty acids (VFA) that are absorbed and digested by the animal to be used as an energy source. Understanding the development of microorganisms within the rumen is of interest due to their effects on host health, energy, performance, and efficiency (Flint and Bayer, 2008; Guan et al., 2008). Evidence suggests that colonization of the calf gastrointestinal tract may occur prior to birth due to the presence of bacteria in the meconium (Alipour et al., 2018). In addition, the establishment of the bacterial communities within the rumen occurs sequentially (Jami et al., 2013; Rey et al., 2014); however, little is known about the relationship and influence of the maternal rumen microbiome on subsequent development of the calf rumen microbiome and VFA production.

Research in humans (Biasucci et al., 2010) and ruminants (Cunningham et al., 2018) suggests that there is a maternal influence on gut microbiome development, including mode of delivery and nutrition. In addition, microbial populations established early in life have the ability to affect the health and performance of the adult animal, suggesting the potential for programming of the microbiome (Abecia et al., 2013). Thus, we hypothesized that the cow maternal rumen microbiome would influence colonization of the calf rumen microbiome. Our objective was to relate the rumen microbiome of the cow prior to parturition (RFC) and at weaning (RFCw) to the calf’s meconium microbiome (M) and calf rumen microbiome at birth (RFd1), day 2 (RFd2), day 28 (RFd28), and weaning (RFNw).

## MATERIALS AND METHODS

Experimental protocols were approved by the Institutional Animal Care and Use Committee of the University of Wyoming (UW).

### Sample Collection

Multiparous Angus crossbred cows (*n* = 10) from the UW beef herd were used in this study. Cows were bred via artificial insemination (AI), their expected calving date was calculated from the AI date, and cows were fed to meet the nutrient requirements through gestation and parturition. Ten to 12 d prior to the expected calving date and at the subsequent weaning, rumen fluid was collected from the cows via oral lavage using methods described by Lodge-Ivey et al. (2009). A flexible vinyl tube was lubricated and passed orally into the rumen where rumen fluid was then collected via suction by an attached syringe. All samples were aliquoted, flash frozen, and stored at −80 °C for subsequent analysis.

Immediately following parturition, meconium and rumen fluid were collected from the calves. Meconium samples were collected using methods described by Alipour et al. (2018). A sterile doubled sheathed equine uterine culture swab was inserted into the calf’s rectum. The cotton tip was exposed and swirled inside the rectum, then retracted into a protective tube prior to removal from the rectum. Rumen fluid was collected immediately prior to meconium via oral lavage. Rumen fluid was collected again 24 h post-parturition (day 2), day 28, and at weaning.

### Microbial DNA Extraction, Library Preparation, and Sequencing

Rumen fluid and meconium samples were processed for 16S rRNA sequencing. Microbial DNA was isolated using bead-beating methods described by Yu and Morrison (2004) and further purified using the QIAamp DNA Stool Kit (Qiagen), quantified by Qubit assay and diluted to 5 ng/µL for library preparation. The hypervariable region V4 of the 16S rRNA gene was sequenced using the Illumina MiSeq platform at the Colorado State University Next Generation Sequencing Laboratory. The first-round PCR was done using primers 515F and 806R. The second-round PCR was done using primers P5F and P7R following Illumina protocol.

### Bioinformatic Analysis

Paired-end sequences were quality filtered, assigned taxonomy, and analyzed for diversity utilizing QIIME2 v. 2019.10 (Bolyen et al., 2019). Taxonomic assignment was completed by aligning to the SILVA 132 database. Shannon index and evenness were calculated for metrics of alpha diversity, and Bray–Curtis distance matrix was utilized for beta diversity. All pairwise comparisons were made using Kruskal–Wallis permutational multivariate analysis of variance. Statistical significance was considered when the false discovery rate adjusted *P*-value (*q*) was ≤0.05.

## RESULTS

### Alpha Diversity

**Figure 1.**
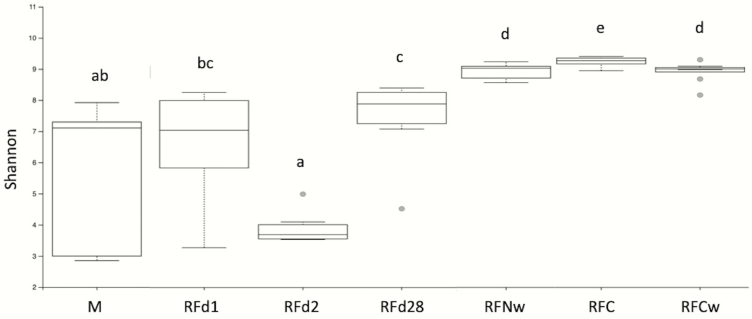
Alpha diversity box plots showing Shannon richness for Meconium (M), calf day 1 rumen fluid (RFd1), calf day 2 rumen fluid (RFd2), calf day 28 rumen fluid (RFd28), calf weaning rumen fluid (RFNw), cow rumen fluid prior to parturition (RFC), and cow rumen fluid at weaning (RFCw). a,b,c,d,e denote differences at the q ≤ 0.05.

**Figure 2.**
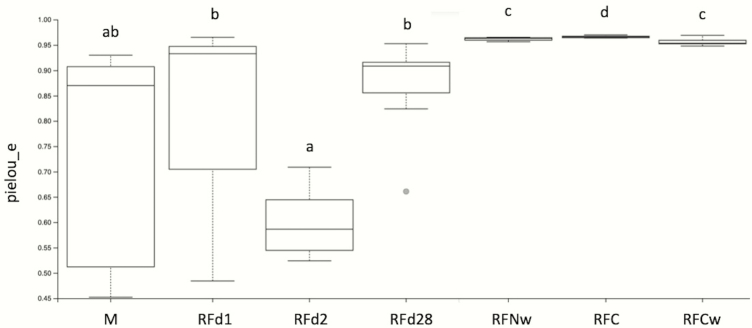
Alpha diversity box plots showing evenness for meconium (M), calf day 1 rumen fluid (RFd1), calf day 2 rumen fluid (RFd2), calf day 28 rumen fluid (RFd28), calf weaning rumen fluid (RFNw), cow rumen fluid prior to parturition (RFC), and cow rumen fluid at weaning (RFCw). a,b,c,d denote differences at the *q* ≤ 0.05.

Shannon diversity accounts for both abundance and evenness of taxa present and is shown in Fig. 1. Meconium differed from RFC, RFCw, RFNw, and RFd28 (*q* ≤ 0.002), but did not differ from RFd1 and RFd2 (*q* ≥ 0.11). Shannon index was significantly greater in RFC than all other samples (*q* ≤ 0.02). Rumen fluid from the cow at weaning differed from all sample types (*q* ≤ 0.02) except for RFNw (*q* = 0.91). Shannon index was significantly greater in RFNw than RFd1, RFd2, and RFd28 (*q* ≤ 0.002). Rumen fluid at day 1 had higher Shannon diversity than RFd2 (*q* = 0.02) but was similar to RFd28 (*q* = 0.14); however, RFd2 was lower compared with RFd28 (*q* = 0.002). Evenness metrics shown in Fig. 2 followed a similar pattern to that of the Shannon diversity, with the exception that M and RFd28 did not differ from each other (*q* = 0.18).

### Beta Diversity

**Figure 3.**
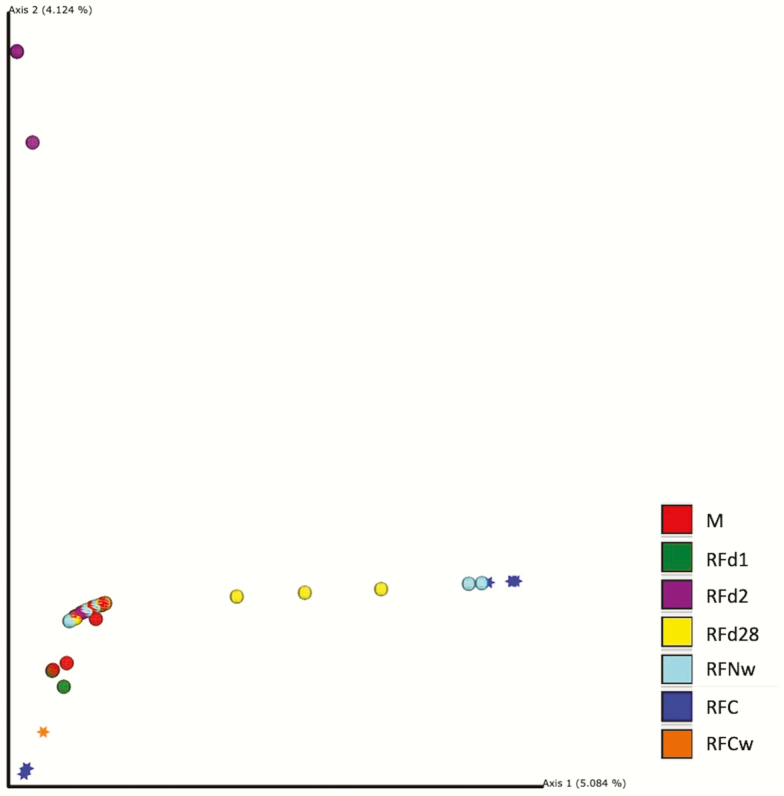
Beta diversity by sample type and date. Principal coordinate analysis plots based on Bray–Curtis dissimilarity matrix for meconium (M), calf day 1 rumen fluid (RFd1), calf day 2 rumen fluid (RFd2), calf day 28 rumen fluid (RFd28), calf weaning rumen fluid (RFNw), cow rumen fluid prior to parturition (RFC), and cow rumen fluid at weaning (RFCw).

The Bray–Curtis dissimilarity matrix is utilized to determine compositional dissimilarities between sample types. Figure 3 indicates that M differed from RFC, RFNw, RFd2, and RFd28 (*q* ≤ 0.01), but did not differ from RFCw and RFd1 (*q* ≥ 0.09). Rumen fluid from the cow prior to parturition differed from RFd1, RFd2, and RFd28 (*q* ≤ 0.03) but was similar to RFCw and RFNw (*q* ≥ 0.08). Rumen fluid from the cow at weaning differed from RFd2 (*q* = 0.04) but was similar to RFNw, RFd1, RFd2, and RFd28 (*q* ≥ 0.46). Beta diversity differed between RFNw and both RFd1 and RFd2 (*q* ≤ 0.01) but was similar to RFd28 (*q* = 0.60). There were differences in composition between RFd1, RFd2, and RFd28 (*q* ≤ 0.04).

## DISCUSSION

It has been suggested that ingestion of amniotic fluid and the associated bacteria resemble that of the meconium and the bacteria within the amniotic fluid may have the potential to influence colonization of the meconium (Ardissone et al., 2014). The similarities in richness, evenness, and composition between meconium and RFd1 could be a result of the ingestion of meconium by the calf during the calving process (Lopez and Bildfell, 1992). Meconium probably has similarities in richness and evenness, but not composition, relative to RFd2 because of differences in nutrient availability at different time points. By RFd2, the rumen microbiome has been exposed to colostrum, milk, and environmental factors leading to different substrate availability to the rumen microbes. The difference in availability of nutrients at day 2 relative to day 1 likely led to shifts in microbial composition of the rumen leading to differences in beta diversity between meconium and RFd2 as well as those found between RFd1 and RFd2. Although the cow rumen microbiome prior to parturition differed from the meconium microbiome, the rumen microbiome of the cow at weaning was similar in composition to the meconium. This could be explained by the research of Alipour et al. (2018) that determined meconium samples resembled the dam oral bacteria. Because ruminant species are known to regurgitate and further chew feed, the cow rumen and oral microbiome are often similar, explaining similarities in phyla between the meconium and rumen fluid samples (Tapio et al., 2016). Hormonal shifts occurring during gestation can lead to changes in gut function and bacterial composition (Koren et al., 2012). Hormonal changes during pregnancy could alter the rumen environment prior to and during parturition explaining the lack of similarities between the meconium and cow rumen fluid prior to parturition. However, the similarities of the meconium and the cow rumen microbiome at weaning could suggest that the early gestation rumen microbiome is more similar to the meconium microbiome. This could indicate that microbial inoculation of the calf in utero begins in early gestation to midgestation; however, further research is needed to understand this relationship.

At the time of weaning, the calf rumen microbiome had increased evenness and richness relative to the RFd1, RFd2, and RFd28; however, RFd28 was similar to the calf at weaning in terms of overall composition. This is indicative of the successive development of the rumen and the stabilization of the microbiome as the animal matures (Jami et al., 2013).

The rumen microbiome of the cow and calf at weaning were similar. This suggests that as the calf matures and consumes feedstuff similar to its dam, their microbiomes become more similar. Beta diversity did not differ between the two mature cow samples, indicating that the compostion of the mature rumen microbiome is stable, but alpha diversity differences were detected. In this instance, cows were sampled at different points of the biological cycle (i.e., weaning vs. late gestation) and were consuming different diets, which could explain the variation in alpha diveristy metrics reported (Kumar et al., 2015).

To our knowledge, this is the first characterization of the meconium microbiome compared with rumen microbiome from birth through weaning and with the maternal rumen microbiome at two different biological stages. With these data, further investigation into key factors affecting in utero colonization are needed. These data may provide a platform to develop programming strategies, thereby impacting host performance long term.

## IMPLICATIONS

Results from this study indicate successional development of the calf rumen microbiome and its relationship to that of the dam rumen microbiome prior to birth and at weaning. These concur with others in the field that the rumen microbiome develops rapidly and in sequential steps following the birthing process. These data provide additional information regarding similarities to the maternal rumen microbiome and can be used to develop hypotheses for the pathway of colonization in the early gut. Further understanding of the maternal influence on the development of the calf rumen microbiome, such as maternal nutrition and reproductive microbiomes, can be used to develop management practices for programming the microbiome and improving host performance.

## References

[CIT0001] Abecia L., Martín-García A. I., Martínez G., Newbold C. J., and Yáñez-Ruiz D. R.. 2013 Nutritional intervention in early life to manipulate rumen microbial colonization and methane output by kid goats postweaning. J. Anim. Sci. 91:4832–4840. doi:10.2527/jas.2012-6142 23965388

[CIT0002] Alipour M. J, Jalanka J., Pessa-Morikawa T., Kokkonen T., Satokari R., Hynönen U., Iivanainen A., and Niku M.. 2018 The composition of the perinatal intestinal microbiota in cattle. Sci. Rep. 8:1–14. doi:10.1038/s41598-018-28733-y 29993024 PMC6041309

[CIT0003] Ardissone A. N., de la Cruz D. M., Davis-Richardson A. G., Rechcigl K. T., Li N., Drew J. C., Murgas-Torrazza R., Sharma R., Hudak M. L., Triplett E. W., et al. 2014 Meconium microbiome analysis identifies bacteria correlated with premature birth. PLoS One 9:e90784. doi:10.1371/journal.pone.0090784 24614698 PMC3948723

[CIT0004] Biasucci G., Rubini M., Riboni S., Morelli L., Bessi E., and Retetangos C.. 2010 Mode of delivery affects the bacterial community in the newborn gut. J. Nutr. 138:1796S–1800S. doi:10.1016/j.earlhumdev.2010.01.004 20133091

[CIT0005] Bolyen E., Rideout J. R., Dillon M. R., Bokulich N. A., Abnet C. C., Al-Ghalith G. A., Alexander H., Alm E. J., Arumugam M., Asnicar Y. F., et al 2019 Reproducible, interactive, scalable and extensible microbiome data science using QIIME 2. Nat. Biotechnol. 37:852–857. doi:10.1038/s41587-019-0209-9 31341288 PMC7015180

[CIT0006] Cunningham H. C., Austin K. J., Powell S. R., Carpenter K. T., and Cammack K. M.. 2018 Potential response of the rumen microbiome to mode of delivery from birth through weaning. Trans. Anim. Sci. 2:S35–S38. doi:10.1093/tas/txy029 PMC7200978 32704733

[CIT0007] Flint H. J., and Bayer E. A.. 2008 Plant cell wall breakdown by anaerobic microorganisms from the mammalian digestive tract. Ann. N. Y. Acad. Sci. 1125:280–288. doi:10.1196/annals.1419.022 18378598

[CIT0008] Guan L. L., Nkrumah J. D., Basarab J. A., and Moore S. S.. 2008 Linkage of microbial ecology to phenotype: Correlation of rumen microbial ecology to cattle’s feed efficiency. FEMS Microbiol. Lett. 288:85–91. doi:10.1111/j.1574-6968.2008.01343.x 18785930

[CIT0009] Jami E., Israel A., Kotser A., and Mizrahi I.. 2013 Exploring the bovine rumen bacterial community from birth to adulthood. ISME J. 7:1069–1079. doi:10.1038/ismej.2013.2 23426008 PMC3660679

[CIT0010] Koren O., Goodrich J. K., Cullender T. C., Spor A., Laitinen K., Bäckhed H. K., Gonzalez A., Werner J. J., Angenent L. T., Knight R., et al. 2012 Host remodeling of the gut microbiome and metabolic changes during pregnancy. Cell 150:470–480. doi:10.1016/j.cell.2012.07.008 22863002 PMC3505857

[CIT0011] Kumar S., Indugu N., Vecchiarelli B., and Pitta D. W.. 2015 Associative patterns among anaerobic fungi, methanogenic archaea, and bacterial communities in response to changes in diet and age in the rumen of dairy cows. Front. Microbiol. 6:781. doi:10.3389/fmicb.2015.00781 26284058 PMC4521595

[CIT0012] Lodge-Ivey S. L., Browne-Silva J., and Horvath M. B.. 2009 Technical note: Bacterial diversity and fermentation end products in rumen fluid samples collected via oral lavage or rumen cannula. J. Anim. Sci. 87:2333–2337. doi:10.2527/jas.2008-1472 19329475

[CIT0013] Lopez A., and Bildfell R.. 1992 Pulmonary inflammation associated with aspirated meconium and epithelial cells in calves. Vet. Pathol. 29:104–111. doi:10.1177/030098589202900202 1378669

[CIT0014] Rey M., Enjalbert F., Combes S., Cauquil L., Bouchez O., and Monteils V.. 2014 Establishment of ruminal bacterial community in dairy calves from birth to weaning is sequential. J. Appl. Microbiol. 116:245–257. doi:10.1111/jam.12405 24279326

[CIT0015] Tapio I., Shingfield K. J., McKain N., Bonin A., Fischer D., Bayat A. R., Vilkki J., Taberlet P., Snelling T. J., and Wallace R. J.. 2016 Oral samples as non-invasive proxies for assessing the composition of the rumen microbial community. PLoS One 11:e0151220. doi:10.1371/journal.pone.0151220 26986467 PMC4795602

[CIT0016] Yu Z., and Morrison M.. 2004 Improved extraction of PCR-quality community DNA from digesta and fecal samples. Biotechniques 36:808–812. doi:10.2144/04365ST04 15152600

